# A systematic review and meta-analysis of microRNAs in the diagnosis of early diabetic kidney disease

**DOI:** 10.3389/fendo.2025.1432652

**Published:** 2025-04-22

**Authors:** Lujie Jiang, Li Li, Ke Zhang, Liping Zheng, Xinhuan Zhang, Yanlian Hou, Mingfeng Cao, Yan Wang

**Affiliations:** ^1^ Department of Endocrinology, The Second Affiliated Hospital of Shandong First Medical University, Taian, Shandong, China; ^2^ Department of Endocrinology, The First People’s Hospital of Taian, Taian, Shandong, China; ^3^ Department of Endocrinology, The First People’s Hospital of Ningyang, Taian, Shandong, China

**Keywords:** microRAN, early diabetic kidney disease, diagnostic tests, systematic review, meta-analysis

## Abstract

**Objective:**

The aim of this study was to comprehensively assess the overall diagnostic value of circulating microRNAs (miRNAs or miRs) as biomarkers for the early diagnosis of diabetic kidney disease (DKD) through Meta-analysis, and to identify potential molecular biomarkers with higher diagnostic value for early DKD.

**Methods:**

The CNKI, Wanfang date, VIP, Pubmed, Embase, Web of Science, and Cochrane Library until January 2024 were searched. Relevant studies associated with the value of miRNAs in the diagnosis of early DKD were selected. Case numbers, sensitivity, and specificity were extracted from the included literature for both the observation and control groups.

**Results:**

Nine studies including 655 cases of early DKD patients and 664 cases as a control group were conducted. The comprehensive sensitivity was 0.76, comprehensive specificity was 0.74, combined positive likelihood ratio was 2.9 and the combined negative likelihood ratio was 0.33, diagnostic odds ratio (DOR) was 9. The summary receiver operating characteristic (SROC) curve was drawn and the area under the curve (AUC) was 0.79. Blood and urine source data were analyzed and showed that urine source miRNA had a higher sensitivity (0.82vs 0.68) and a higher DOR (10.5vs 8.2) than blood source miRNA.

**Conclusion:**

MiRNAs may serve as promising noninvasive biomarkers for the early diagnosis of DKD. The diagnostic value of miRNAs in urine samples may be higher than that in blood samples. The combined detection of some miRNAs or other clinical indicators can enhance the accuracy of early DKD diagnosis.

**Systematic Review Registration:**

https://osf.io, identifier DOI: 10.17605/OSF.IO/FC6DK.

## 1 Introduction

Diabetes mellitus (DM) is a common chronic metabolic disease, and as the disease progresses, it leads to various chronic complications such as macrovascular, microvascular, and multi-organ damage, significantly affecting the quality of life and survival time of patients. Diabetic kidney disease (DKD) is one of the most common microvascular complications in diabetic patients, and it is a kind of progressive nephropathy, which often leads to an increased risk of cardiovascular diseases. Without systematic diagnosis and treatment, DKD is highly likely to progress to end stage renal disease(ESRD). ESRD is one of the main causes of death in DKD patients (1, 2).

The early stage of DKD is the urine microalbumin (UMA) stage, and the diagnosis depends on the detection of urinary albumin excretion rate (UAE). However, UAE lacks sensitivity and specificity in detecting the early onset of DKD, and is easily influenced by sports, infection, pregnancy, and other factors. Moreover, it cannot effectively predict the progression of DKD (3). Currently, the gold standard for diagnosing DKD and assessing its severity is renal biopsy. However, due to its invasive nature and high cost, most patients are reluctant to undergo this procedure (4).

MiRNAs are short non-coding RNA molecules composed of about 22 nucleotides. They regulate gene expression at the post-transcriptional level, mediating gene silencing and inhibiting protein synthesis (5). MiRNAs play roles in cell growth, apoptosis, proliferation, embryonic development, and tissue differentiation, and are now widely studied as biomarkers for the diagnosis, treatment, and prognosis of various diseases (6–8). Some miRNAs, such as miR-216a and miR-377, exhibit abnormal expression levels in the early stages of DKD, with their expression changing as the disease progresses (9). The abnormal expression of some miRNAs in diabetic patients may predate the appearance of albuminuria (10). MiRNAs are stable, present in various biological fluids (e.g., blood, urine), and are easily obtained and detected. Therefore, miRNAs may become new biological markers for the early diagnosis of DKD.

At present, few studies assessed the accuracy of miRNAs in the early diagnosis of DKD, and their diagnostic value in early DKD remains inadequately evaluated. In this study, we aim to evaluate the overall diagnostic value of miRNAs for early DKD by analyzing the research results in the existing literature and using statistical software and try to find sensitive and specific molecular markers for the early diagnosis of DKD.

## 2 Methods

### 2.1 Literature retrieval strategy

The CNKI, Wanfang, Vip databases, PubMed, Embase, and Cochrane Library were searched to obtain relevant literature published up to Jan 2024. We used the keywords and subjects both in Chinese and English as follows:”Diabetic nephropathy、Diabetic Kidney Disease、Diabetic Glomerulosclerosis、Kimmelstiel-Wilson Syndrome、microRNA*、miRNA*、Primary MicroRNA、Small Temporal RNA、Diagnosis、Sensitivity、Specificity、Accuracy/Validity”.

### 2.2 Inclusion criteria

The inclusion criteria were as follows: (1) Studies related to the early diagnosis of DKD by miRNAs. (2) The observation group was patients with early DKD (excluding diabetes combined with other nephropathies), and the control group was patients with simple diabetes (without DKD), and the diagnosis of diabetes all met the current diabetes guideline diagnostic criteria, with no restrictions on age, gender, or ethnicity. (3) The number of cases, sensitivity (Sen) and specificity (Spe) of the observation group and the control group can be obtained, and table data could be calculated, including true positives (TP), false positives (FP), false negatives (FN), true negatives (TN). (4) The patients in the observation group were clinically diagnosed with early DKD (30mg/24h < UAE < 300mg/24h or 20ug/min < UAE < 200ug/min). (5) The detection methods and reagent sources of miRNAs in the literature are clear. (6) Literature in Chinese or English. The exclusion criteria were as follows: (1) Does not meet the inclusion criteria; (2) Simple descriptive study without control group; (3) Animal research or cell research; (4) Literature review, meetings, letters, and case reports; (5) Articles that could not be obtained complete data. (6) Repeated literature.

### 2.3 Data extraction and management

According to the inclusion and exclusion criteria, the literature was strictly screened and data were extracted from the literature independently. The main data extracted included the following: first author, publication time, research country, target miRNAs, miRNA sample source, detection method, sample size, sensitivity, specificity, the four-grid table data (including TP, FP, FN, TN). If only the ROC curve was provided in the included literature and specific values for sensitivity and specificity were not available, the Data can be obtained through Get Data software analysis.

### 2.4 Quality assessment

QUADAS-2 in RevMan 5.4 was used to evaluate the quality of the included studies, covering four domains: Patient Selection, Index Test, Reference Standard, and Flow and Timing. The clinical applicability of the enrolled patients, the index test, and the reference standard was assessed separately by answering “yes”, “no”, or “ unclear “ to the relevant landmark questions included in each section (11).

### 2.5 Synthesis and statistical analysis

Stata16 and Meta-Disc 1.4 software were used for data analysis. First, the Q-test and the I^2^-test were used to examine the heterogeneity of the studies: if I^2^ < 50%, the fixed-effect model was used, and if I^2^ > 50%, the random-effect model was used. Subsequently, the extracted data were statistically analyzed, including sensitivity, specificity, positive likelihood ratio (PLR), negative likelihood ratio (NLR) and diagnostic odds ratio (DOR). Simultaneously, a summary receiver operating characteristic (SROC) curve was drawn and the area under the curve (AUC) was calculated. AUC values were 0.5-0.7, 0.7-0.9, and 0.9-1.0, represent low, moderate, and high diagnostic accuracy, respectively. Then Meta-Disc1.4 software was used to calculate Spearman correlation coefficient to evaluate the heterogeneity caused by threshold effect: if *P* > 0.05, there was no threshold effect, and all indicators can be combined; If *P* < 0.05, a threshold effect existed, and the research indicators could not be combined. The sources of heterogeneity were discussed by regression analysis and subgroup analysis. Sensitivity analysis was also conducted to explore the stability and reliability of all included literature. Finally, Deek’s funnel plots were used to test for publication bias, which was present if *P* < 0.10.

## 3 Results

### 3.1 Summary of searches and filter results

**Figure 1:**
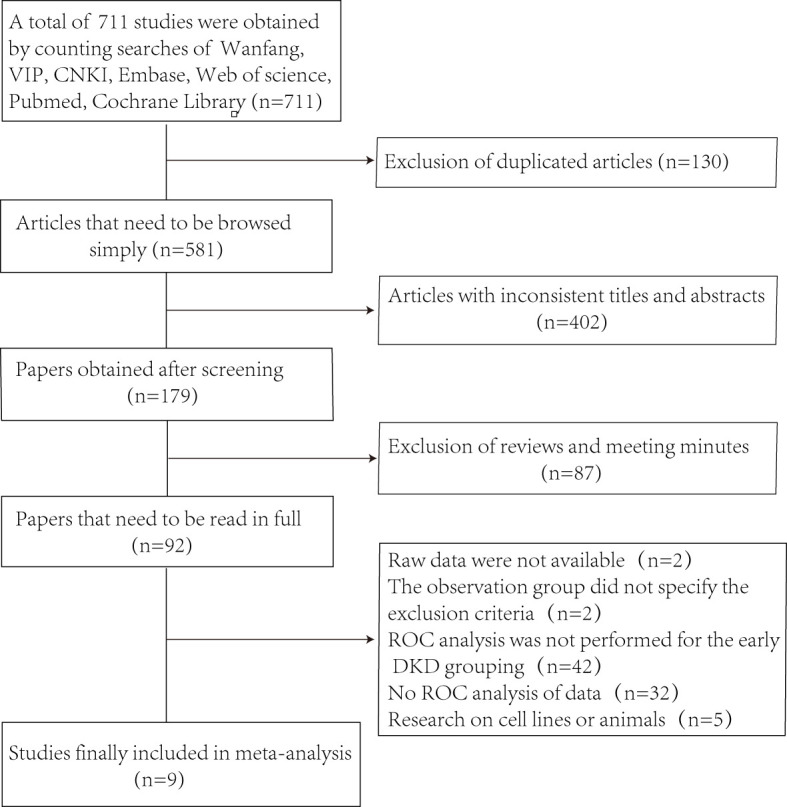
Articles screening flow chart.

**Figure 1** shows the selection process of the studies.Through searching the database, 711 studies were obtained and 581 articles were retained after excluding 130 duplicates. By browsing the titles and abstracts, 402 articles were excluded with inconsistent themes, and 87 articles such as reviews, conferences, and reports. The next step of full-text reading excluded 92 papers. In the end, we obtained 9 studies (12–20), which included 655 patients with early DKD and 664 controls.

### 3.2 The basic characteristics of articles

The basic characteristics of the included literature were shown in **Table 1**. The following data were extracted from the studies: first author, publication date, country of study, target miRNA, sample source, miRNA detection method, sample size of observation and control groups, TP, FP, FN, and TN. Two studies only provided ROC curves and AUC values, and sensitivity and specificity were obtained using the Get Data software.

**Table 1:** Basic characteristics of 9 included articles.

Group	First Author	Publication Date	Country	Target MiRNA	MiRNA’s Sample source	Method for Detect MiRNA	Sample size	Observation group	Control Group	TP	FP	FN	TN
1	Yijie Jia (a-2) (19)	2016	China	MiR-192	Urine	qRT-PCR	60	30	30	26	11	4	19
2	Yijie Jia (b-2) (19)	2016	China	MiR-194	Urine	qRT-PCR	60	30	30	11	3	19	27
3	Yijie Jia (b-2) (19)	2016	China	MiR-215	Urine	qRT-PCR	60	30	30	23	13	7	17
4	Xinyi Yang (a-1) (18)	2018	China	MiR-192	Serum	qRT-PCR	179	87	92	62	32	25	60
5	Xinyi Yang (a-1) (18)	2018	China	MiR-192	Urine	qRT-PCR	179	87	92	85	29	2	63
6	P. Prabu(a-2) (20)	2019	India	let-7i+ miR-24+miR-27+miR-15	Urine	qRT-PCR	80	40	40	32	14	8	26
7	Qianhu Wang (a-1) (13)	2018	China	MiR-21	Plasma	PCR	340	210	130	114	41	96	89
8	Junyi He (a-1) (15)	2018	China	miR-233-3p	Plasma	PCR	110	55	55	26	14	29	41
9	Boxun Luo (a-1) (16)	2020	China	MiR-192	Plasma	qRT-PCR	159	98	61	68	12	30	49
10	Boxu Luo (b-1) (16)	2020	China	MiR-29c	Plasma	qRT-PCR	159	98	61	62	17	36	47
11	Boxun Luo (c-1) (16)	2020	China	MiR-192+ MiR-29c	Plasma	qRT-PCR	159	98	61	90	7	8	54
12	Qiuli Jia (a-1) (17)	2022	China	MiR-21	Plasma	qRT-PCR	122	52	70	40	17	12	53
13	Min Xiong (a-1) (12)	2022	China	MiR-155	Serum	qRT-PCR	101	37	64	27	16	10	48
14	Lihua Hong (a-1) (14)	2022	China	MiR-29c	Serum	qRT-PCR	168	46	122	39	24	7	98

Tp, True Positive; Fp, False Positive; Fn, False Negative; Tn, True Negative.

a, b, c are used to distinguish different microRNAs within the same literature.

1 represents microRNAs from blood sources, and 2 represents microRNAs from urine sources.

### 3.3 Quality evaluation results

**Figure 2:**
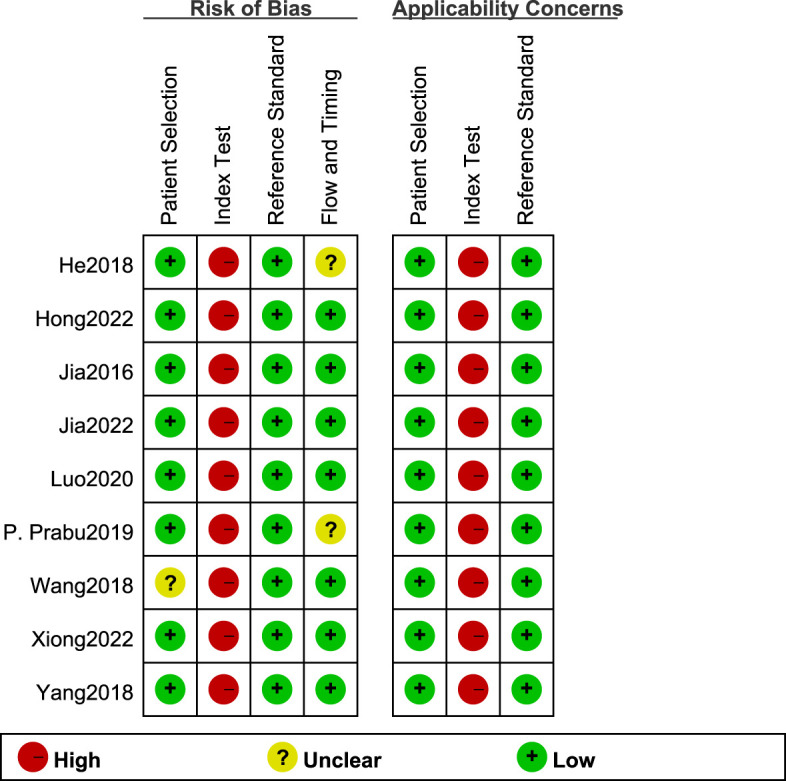
Quality evaluation of 9 included documents.

The QUADAS-2 scale in RevMan5.4 was used to evaluate the quality of the eight articles (**Figure 2**). All study types were clinical diagnostic studies, and the reference diagnostic criteria for all experiments were diabetic patients with combined 30 mg/24h < UAE < 300 mg/24h or 20ug/min < UAE < 200ug/min. Among them, Wang’s study (13) did not indicate the type of diabetes in the patients included in the Observation group, which resulted in an unclear risk of bias in the area of case selection. His study (15) did not include all study participants in the data analysis for reasons not indicated, which may cause uncertainty in the part of flow and timing. The high risk of bias of the tests to be evaluated can be seen in the **Figure 2**, which may be related to the implementation of the tests, the different processes, and the lack of predefined thresholds. Since studies on circulating miRNAs for the diagnosis of early DKD are rare, most studies derived optimal diagnostic thresholds based on ROC curves, and there was no unified diagnostic threshold standard. Overall, the quality of the included studies was satisfactory.

### 3.4 Threshold effect analysis

The Meta-Disc1.4 software was used to calculate the Spearman correlation coefficient between the logarithm of sensitivity and the (1-specific) logarithm was 0.157 (r = 0.154, *P* > 0.05), indicating that there was no significant heterogeneity of threshold effects between this study. The SROC curves did not show a “shoulder-arm” feature, which further proved that there was no threshold effect in this study.

### 3.5 Statistical analysis results

**Figure 3:**
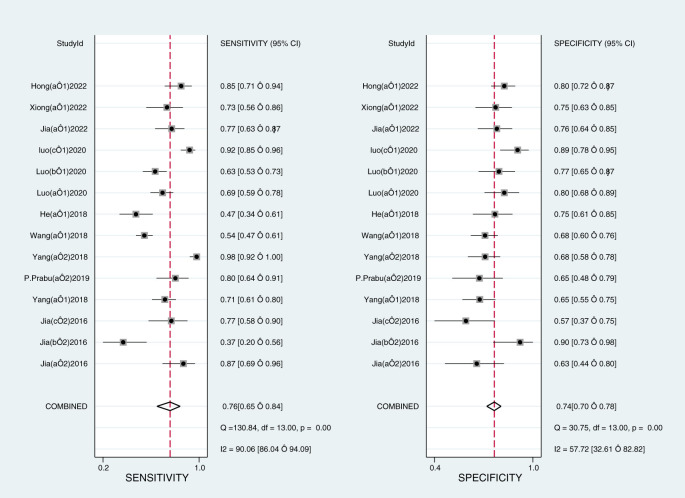
Forest plot of sensitivity and specificity of microRNAs in the early diagnosis of DKD.

**Figure 4:**
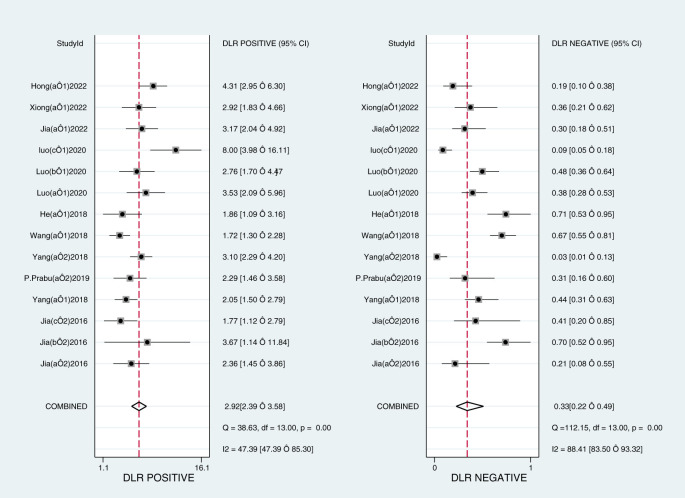
Forest plot of DLR Positive and DLR Negative of microRNAs in the early diagnosis of DKD.

**Figure 5:**
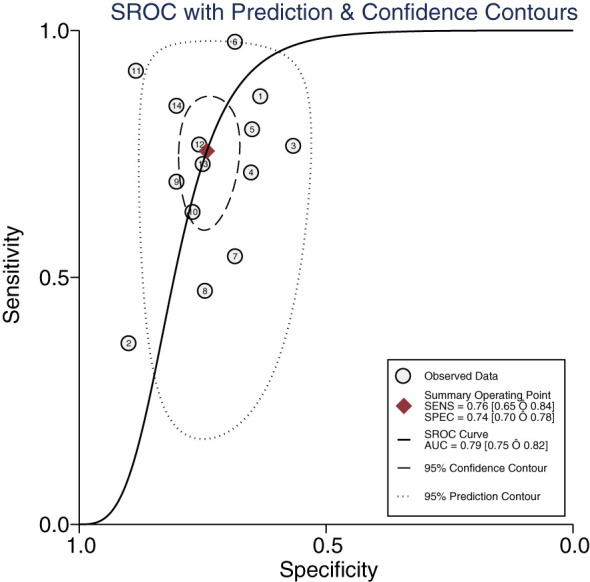
The summary receiver operating characteristic curve of miRNAs in the early diagnosis of DKD.

The Midas module in Stata16 software was used for the meta-analysis. The Q-test and I^2^-test both suggested heterogeneity among the included literature, so the random effects model in Stata16 software was chosen for the combined analysis of sensitivity, specificity, PLR, NLR, and DOR. Overall diagnostic value of miRNA in early DKD diagnosis: The comprehensive sensitivity was 0.76 (95% CI: 0.65, 0.84) and the comprehensive specificity was 0.74 (95% CI: 0.70, 0.78) (**Figure 3**), the PLR was 2.9 (95% CI: 2.4, 3.6) and the NLR was 0.33 (95% CI: 0.22, 0.49) (**Figure 4**), DOR was 9 (95% CI: 5, 15). A generalized SROC curve was plotted with an AUC of 0.79 (95% CI: 0.75, 0.82) (**Figure 5**). It can be seen that all the indicators suggest a high overall efficacy of miRNAs detection for the early diagnosis of DKD.

### 3.6 Subgroup analysis

**Figure 6:**
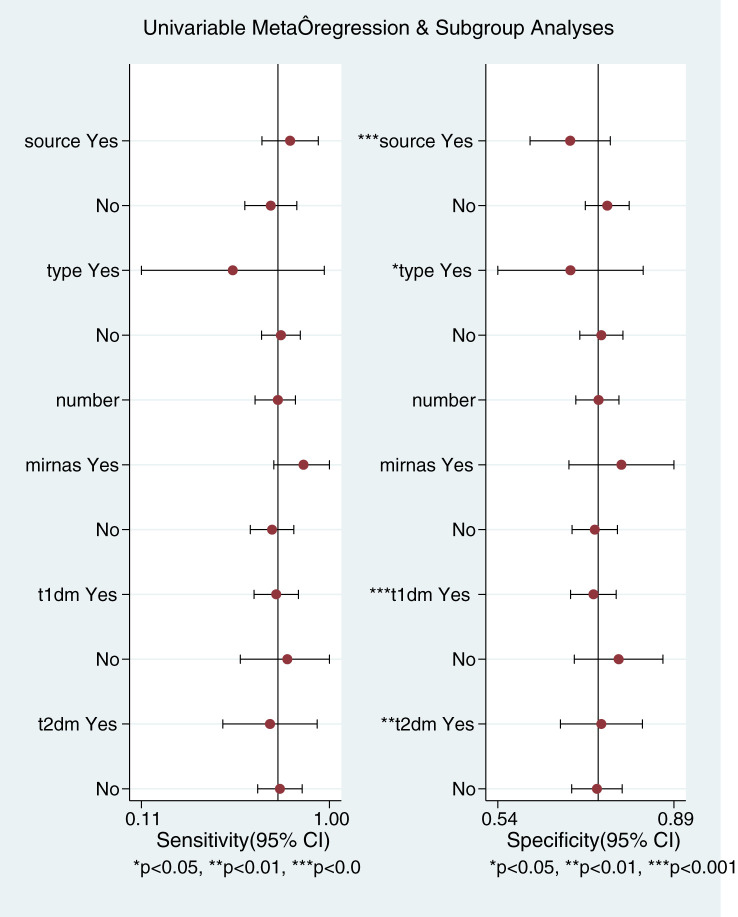
Regression analysis.

This Meta-analysis included studies with a high degree of heterogeneity, with an I^2^ of more than 50%, subgroup analyses and meta-regression analyses were performed for single factors. Heterogeneity in the studies may arise from factors such as source of miRNAs, number of miRNAs, sample size, type of diabetes, and regression analyses were performed on these factors (**Figure 6**). It can be seen that the source of the sample and the type of diabetes were the main sources of heterogeneity (*P*<0.01). Then Meta Disc1.4 was used for subgroup analysis: (1) Urine miRNA (Sen 0.82, 95%CI: 0.76, 0.87) may be more efficient than blood miRNA (Sen 0.68, 95%CI: 0.64, 0.71) in diagnosing early DKD (**Table 2**). (2) Blood sample sources were further categorized into serum and plasma, and the results (**Table 3**) showed that plasma-derived miRNAs compared to serum-derived miRNAs which had higher AUC (0.92 vs. 0.79), higher Sen (71% vs. 65%), and higher Spe (80% vs. 73%), as well as higher DOR (10.0 vs. 7.0). (3) Data were analyzed for the T2DM group (**Table 4**): AUC = 0.82, Sen was 0.75 (95% CI: 0.71, 0.78), Spe was 0.73 (95% CI: 0.70, 0.77), PLR was 2.7 (95% CI: 2.2, 3.3), NLR was 0.33 (95% CI: 0.23, 0.50), DOR = 9.3 (95% CI: 5.2, 16.9). Data analysis of T1DM group showed that the Sen was 0.80 (95% CI: 0.70, 0.88), the Spe was 0.79 (95% CI: 0.72, 0.84), the PLR was 3.6(95% CI: 2.5, 5.3) and the NLR was 0.27 (95% CI: 0.14, 0.52), and DOR = 13.6 (95% CI: 5.0, 37.4). AUC could not be obtained due to the small sample in the T1DM group.

**Table 2:** Analysis results of sample source subgroups.

Factor	Subgroup	Number	Sen(95%CI)	Spe (95%CI)	PLR(95%CI)	NLR(95%CI)	DOR(95%CI)	AUC
**Sample**	blood	9	0.68(0.64,0.71)	0.75(0.72,0.78)	2.8(2.2,3.8)	0.37(0.26,0.53)	8.2(4.3,15.6)	0.83
**Sources**	urine	5	0.82(0.76,0.87)	0.69(0.62,0.75)	2.5(2.0,3.1)	0.25(0.09,0.75)	10.5(3.9,28.6)	0.80

**Table 3:** Analysis results of blood sample subgroups.

Factor	Subgroup	Number	Sen(95%CI)	Spe (95%CI)	PLR(95%CI)	NLR(95%CI)	DOR(95%CI)	AUC
**Sample**	serum	5	0.65(0.61,0.70)	0.73 0.69,0.76)	2.6 (1.9,3.7)	0.38(0.24,0.61)	7.0(3.3,15.0)	0.79
**Sources**	plasma	5	0.71(0.65,0.75)	0.80(0.75,0.85)	3.4(2.0, 5.8)	0.35(0.19,0.66)	10.0(2.9,35.1)	0.92

**Table 4:** Analysis results of diabetes type subgroups.

Factor	Subgroup	Number	Sen(95%CI)	Spe (95%CI)	PLR(95%CI)	NLR(95%CI)	DOR(95%CI)	AUC
**Sample**	T1DM	2	0.80(0.70,0.88)	0.79(0.72,0.84)	3.6(2.5,5.3)	0.27(0.14,0.52)	13.6(5.0,37.4)	–
**Sources**	T2DM	11	0.75(0.71,0.78)	0.73(0.70,0.77)	2.7(2.2,3.3)	0.33(0.23,0.50)	9.3 (5.2,16.9)	0.82

### 3.7 Sensitivity analysis

**Figure 7:**
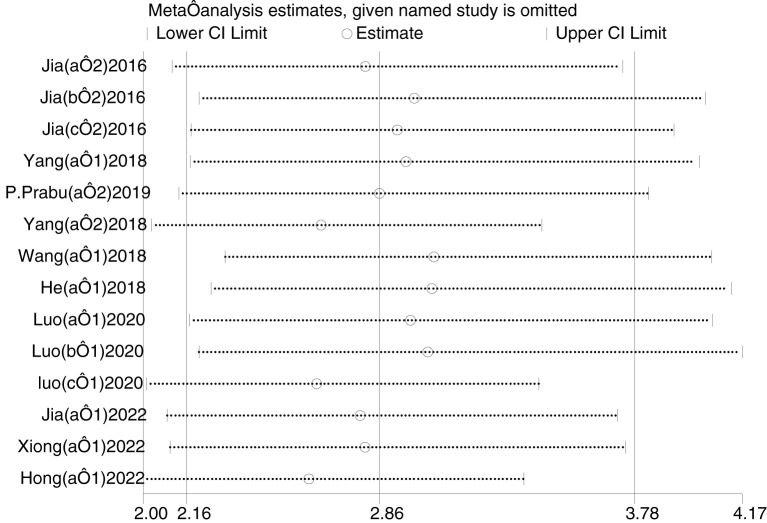
Sensitivity analysis.

Sensitivity analysis was performed using Stata 16 (**Figure 7**). After excluding individual studies each time and regrouping the remaining studies, the results did not change significantly, indicating that the sensitivity of the included studies was lower and the results were more robust and credible.

### 3.8 Publication bias

**Figure 8:**
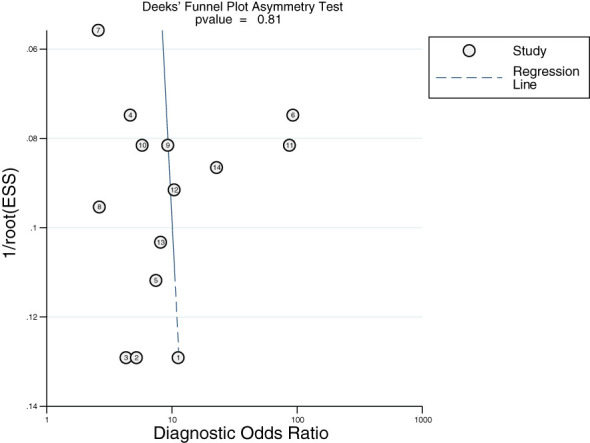
Deek’s funnel.

Deek’s funnel plot was plotted using Stata 16. As shown in **Figure 8**, there was no obvious publication bias in the included studies (*P* > 0.10).

## 4 Discussion

Diabetes mellitus (DM) has a complex pathogenesis, and as the disease progresses, various chronic complications arise, and diabetic kidney disease (DKD) is one of the most serious microvascular complications in diabetic patients. UMA was present in some T2DM patients when they were diagnosed with diabetes (21), early identification and treatment of diabetic nephropathy can slow the progression of DKD, reduce the high mortality and morbidity associated with decreased kidney function, and reduce the expenditure of medical resources (22). Positive urinary albumin is not a characteristic change of DKD (23, 24). Compared with other kidney diseases, renal pathological changes in the early DKD stage lack specificity, and hypertension and diabetes can also lead to thickening of glomerular basement membrane (25). Moreover, The operation of renal biopsy is risky and expensive.

Circulating miRNAs have emerged as potential diagnostic biomarkers for cancer, serving as independent prognostic markers and therapeutic targets for breast cancer risk stratification (26, 27), as well as diagnostic and therapeutic targets for liver-related diseases (28, 29). MiRNAs play an important role in the progression of DKD, and their expression levels appear to change accordingly as DKD progresses (30–32). MiRNAs exist stably in biological fluids and are easy to obtain, which makes it convenient to repeat the detection. Currently, quantitative real-time PCR (RT- qPCR) is used to detect miRNAs, which has the advantages of high sensitivity, good reproducibility, and accurate quantification. Therefore, by measuring the changes in miRNAs, it can be used as a simple method to identify the early stages of DKD.

Our study primarily focused on the diagnostic value of miRNAs in early diagnosis of DKD. Meta-analysis showed that the overall sensitivity was 0.76 and the overall specificity was 0.74. The PLR was 2.9, suggesting that the positive rate of miRNAs in early diabetic nephropathy patients was 2.9 times higher than that in DKD patients alone. The DOR was 9 and the NLR was 0.33, suggesting that miRNAs possess strong diagnostic potential for the early stages of DKD. In this study, the AUC was 0.79, which suggests that miRNAs have good diagnostic value for the early stages of DKD.

From the subgroup analyses, the overall diagnostic value of miRNAs in urine samples for the early stages of DKD may be higher than that of blood samples, suggesting that we should pay more attention to urine samples in future studies.Urine samples are not only easier to collect but also less susceptible to external factors compared to blood samples. MiRNAs are stably present in urine exosomes and can be detected by RT-qPCR. Currently, the use of abnormally expressed miRNAs in patients’ urine to diagnose DKD is a hot research topic. It has been confirmed that a variety of miRNAs are abnormally expressed in urine exosomes of patients with DKD (33).

In this study, miR-192+miR-29c was included for the combined early diagnosis of DKD which sensitivity and specificity were 0.92 and 0.89, respectively, which were higher than those of the single miRNAs for diagnosing the early stages of DKD; however, due to the small number of studies on the combined diagnosis, it was not possible to analyze the data merging and plot the SROC curve. Therefore, further expansion of the study and sample size is needed for validation in future studies. In addition, the combination of miRNA with other indicators is also valuable in the early diagnosis of DKD. Several studies have shown that the diagnostic efficacy of miRNA in combination with other indicators is higher than that of miRNA alone, and urinary neutrophil gelatinase-associated lipocalin (NGAL) has been widely investigated as a marker for acute tubular injury (34) and chronic kidney disease of various etiologies (35, 36). The combination of serum miRNA-21, Smad1, and urinary NGAL has significantly higher sensitivity and specificity for diagnosing early-stage DKD than the individual tests (13). Long non-coding RNAs (lncRNAs) also belong to the non-coding RNA family and are involved in the development and progression of many acute and chronic kidney diseases, including DKD (37). The expression of long noncoding RNA metastasis-associated lung adenocarcinoma transcript 1 (LncRNA MALAT1) was up-regulated in the serum of the early stages of DKD patients, and its expression level was positively correlated with the progression of DKD, and the combination of serum MALAT1 and miR-29 can further improve the diagnostic efficacy of early DKD (38).

The limitations of this study include the following aspects: (1) At present, there are few studies on the early diagnosis of DKD using miRNAs. Some studies derive the optimal diagnostic threshold based on the ROC curve. The risk of bias in index tests is high because there are no standardized criteria for diagnostic thresholds. (2) The number of cases included in the studies was relatively small, and there was significant heterogeneity in the methodological differences and biological effects of miRNAs. Therefore, meta-analysis should be performed on a larger sample size and higher-quality studies to validate and improve the results of this study. (3) Although there was no publication bias in the included literature and the retrieved literature was screened strictly according to inclusion and exclusion criteria, heterogeneity could not be eliminated. No clear source of heterogeneity was identified by subgroup analysis. Possible reasons for the heterogeneity of miRNAs in the early stages of DKD may be as follows: type of diabetes in the experimental group, exclusion criteria for the experimental group, study design, inconsistent selection of assays and reagents, and different cutoff values used. Generally speaking, the results of publication bias test included in this study are acceptable.

In summary, this study concludes that miRNAs can be used as promising molecular biomarkers for the early diagnosis of DKD, and have high value in clinical diagnosis. However, there are still few diagnostic studies on the altered expression levels of miRNAs in early-stage DKD patients, and the lack of data validation from diverse global populations and large sample sizes poses limitations on the quality and quantity of the included literature. These findings need to be further confirmed through more extensive and higher-quality studies.

## 5 Conclusion

The diagnostic value of miRNAs in urine samples may be higher than that in blood samples. The combined detection of some miRNAs or other clinical indicators can improve the accuracy of early diagnosis for DKD. MiRNAs may be promising noninvasive biomarkers for the early diagnosis of DKD.

## Data Availability

The datasets presented in this study can be found in online repositories. The names of the repository/repositories and accession number(s) can be found in the article/supplementary material.
